# Association of Cervical and Lumbar Paraspinal Muscle Composition Using Texture Analysis of MR-Based Proton Density Fat Fraction Maps

**DOI:** 10.3390/diagnostics11101929

**Published:** 2021-10-18

**Authors:** Egon Burian, Edoardo A. Becherucci, Daniela Junker, Nico Sollmann, Tobias Greve, Hans Hauner, Claus Zimmer, Jan S. Kirschke, Dimitrios C. Karampinos, Karupppasamy Subburaj, Thomas Baum, Michael Dieckmeyer

**Affiliations:** 1 Department of Diagnostic and Interventional Neuroradiology, School of Medicine, Klinikum Rechts der Isar, Technical University of Munich, 81675 Munich, Germany; Edoardo.becherucci@tum.de (E.A.B.); nico.sollmann@tum.de (N.S.); claus.zimmer@tum.de (C.Z.); jan.kirschke@tum.de (J.S.K.); thomas.baum@tum.de (T.B.); michael.dieckmeyer@tum.de (M.D.); 2 Department of Diagnostic and Interventional Radiology, School of Medicine, Klinikum Rechts der Isar, Technical University of Munich, 81675 Munich, Germany; Daniela.junker@tum.de (D.J.); dimitrios.karampinos@tum.de (D.C.K.); 3 TUM-Neuroimaging Center, Klinikum Rechts der Isar, Technical University of Munich, 81675 Munich, Germany; 4 Department of Diagnostic and Interventional Radiology, University Hospital Ulm, 89081 Ulm, Germany; 5 Department of Neurosurgery, University of Munich, 81377 Munich, Germany; tobias.greve@med.uni-muenchen.de; 6 Institute of Nutritional Medicine, School of Medicine, Klinikum Rechts der Isar, Technical University of Munich, 80992 Munich, Germany; hans.hauner@tum.de; 7 Engineering Product Development (EPD) Pillar, Singapore University of Technology and Design (SUTD), Singapore 487372, Singapore; subburaj@sutd.edu.sg

**Keywords:** magnetic resonance imaging, quantitative imaging, proton density fat fraction, muscle composition, paraspinal muscle, texture analysis

## Abstract

In this study, the associations of cervical and lumbar paraspinal musculature based on a texture analysis of proton density fat fraction (PDFF) maps were investigated to identify gender- and anatomical location-specific structural patterns. Seventy-nine volunteers (25 men, 54 women) participated in the present study (mean age ± standard deviation: men: 43.7 ± 24.6 years; women: 37.1 ± 14.0 years). Using manual segmentations of the PDFF maps, texture analysis was performed and texture features were extracted. A significant difference in the mean PDFF between men and women was observed in the erector spinae muscle (*p* < 0.0001), whereas the mean PDFF did not significantly differ in the cervical musculature and the psoas muscle (*p* > 0.05 each). Among others, Variance(global) and Kurtosis(global) showed significantly higher values in men than in women in all included muscle groups (*p* < 0.001). Not only the mean PDFF values (*p* < 0.001) but also Variance(global) (*p* < 0.001), Energy (*p* < 0.001), Entropy (*p* = 0.01), Homogeneity (*p* < 0.001), and Correlation (*p* = 0.037) differed significantly between the three muscle compartments. The cervical and lumbar paraspinal musculature composition seems to be gender-specific and has anatomical location-specific structural patterns.

## 1. Introduction

Muscle structure and composition change over the human life span. Not only increasing body mass index (BMI) and age are associated with structural alterations, including fatty infiltration and atrophy, but also katabolic conditions, such as sarcopenia and cachexia [1,2,3,4,5]. There is evidence for connections between the overall muscle quality and muscular performance [6]. Further, the loss of functional muscle tissue due to fatty replacement leads to increasing disability and mortality [7]. Recently, it has also been shown that preoperative sarcopenia is associated with poorer overall survival in pancreatic cancer patients following pancreaticoduodenectomy [8]. Thus, non-invasive muscle status assessments beyond quantification of mere volumetric changes are of increasing interest with regard to personalized medicine and an individual, multi-parametric patient evaluation.

In clinical practice, established imaging methods such as dual-energy x-ray absorptiometry (DXA) and computed tomography (CT) are used to assess muscle status [9]. In certain settings, more advanced imaging techniques such as single-voxel proton magnetic resonance spectroscopy (MRS) and chemical shift encoding-based water–fat magnetic resonance imaging (CSE-MRI) are important diagnostic tools to extract biomarkers such as the proton density fat fraction (PDFF) or analyze the chemical structure of fatty acids within different tissue types [10]. There are plenty of data to support the role of water–fat MRI in the context of muscle composition analysis in healthy adults [11,12,13,14,15,16,17]. Recently, studies using texture analysis (TA) of PDFF maps derived from CSE-MRI showed promising results with regard to structural changes of vertebral bone marrow and paravertebral musculature with good functional correlation [18,19]. As demonstrated in these studies, TA can generate novel information which captures structural changes in osseous compartments and musculature beyond the mean PDFF [18,19].

However, there is still a lack of normative studies investigating the structural composition and morphological association of large muscle groups. The paravertebral musculature exhibits age- and gender-specific degeneration patterns, which are relevant in the context of musculoskeletal disorders [2,20,21]. The comparison of structural muscular changes with regard to gender and anatomical location has the potential to contribute valuable information on muscle (patho) physiology.

Therefore, this study investigated the associations of different cervical and lumbar paravertebral muscle groups based on TA of PDFF maps to identify gender- and region-specific structural patterns.

## 2. Materials and Methods

### 2.1. Subjects

Seventy-nine volunteers with self-reported absence of any musculoskeletal or metabolic diseases (male = 25, female = 54) were recruited from a large cohort recruited for evaluation of determinants of basal metabolic rate [22]. The study protocol and procedures were approved by the local ethics committee of the Faculty of Medicine. Inclusion criteria were: age of 18 years or older, no history of severe diseases or surgery within the last three months, no acute physical impairment. Exclusion criteria were standard contraindications for MRI (implanted pacemaker or MRI-incompatible implants or devices). All subjects provided written informed consent prior to study inclusion.

### 2.2. Magnetic Resonance Imaging

All subjects underwent MRI at 3 Tesla (Ingenia, Philips Healthcare, Best, Netherlands) using the built-in 12-channel posterior coil and a 16-channel anterior coil (dStream Torso coil, Philips Healthcare, Best, Netherlands). Subjects were positioned head-first in supine position.

The imaging protocol comprised an axially prescribed, six-echo three-dimensional (3D) spoiled gradient echo sequence in two stacks for chemical shift encoding-based water–fat separation at the cervical and lumbar spine, respectively.

The dedicated sequence parameters were set as follows: field of view (FOV) = 400 × 300 × 140 mm^3^ (RL × AP × FH), acquisition matrix size = 268 × 201 × 93 mm^3^, acquisition voxel size = 1.5 × 1.5 × 1.5 mm^3^, SENSE with reduction factor = 2.5 × 1.0 mm^3^ (AP × FH, phase × slice). The six echoes were acquired in a single TR using non-flyback (bipolar) read-out gradients; cervical stack: repetition time (TR)/echo time (TE_min_)/echo time step (ΔTE) = 8.2/1.24/1.0 ms, number of signal averages (NSA) = 3, resulting scan time = 4:16 min; lumbar stack: TR/TE_min_/ΔTE = 12.0/1.24/1.0 ms, NSA = 2, resulting scan time = 2:01 min.

### 2.3. Muscle Fat Quantification

The gradient echo imaging data were processed online using the fat quantification routine of the MRI vendor (Philips Healthcare, Best, The Netherlands). After phase error correction, complex-based water–fat decomposition was performed using a precalibrated seven-peak fat spectrum model with a single T2 * [23,24]. The PDFF maps were generated voxel by voxel by computing the ratio of the fat signal and the sum of fat and water signals. Mean PDFF values were computed by averaging the PDFF maps over the ROIs of the segmented muscle groups. Segmentations were performed by a radiologist using the free open-source software Medical Imaging Interaction Toolkit (MITK, developed by the Division of Medical and Biological Informatics, German Cancer Research Center, Heidelberg, Germany; www.mitk.org, accessed on 18 October 2021).

**Figure 1:**
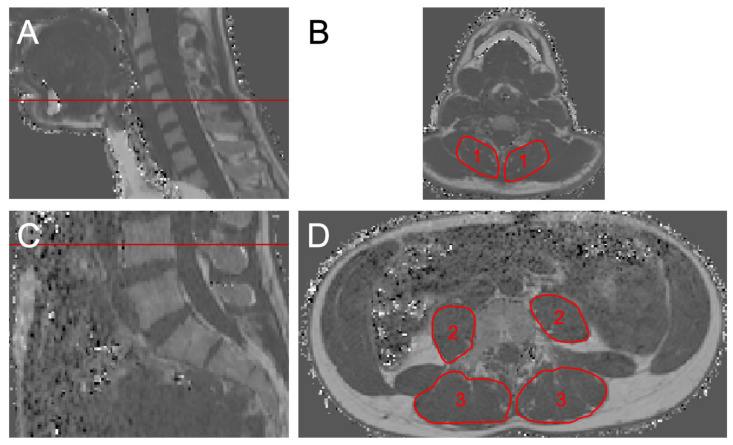
Segmentation example. (**A**,**B**) The segmented CE (left and right multifidus, semispinalis, and spinalis cervicis muscles (1)) at the level of C5. (**C**,**D**) The PS (2) and ES and the multifidus muscles (3) at the level of L4.

The cervical musculature (CE), the erector spinae muscle (ES) and the psoas muscle (PS) were manually segmented bilaterally in the PDFF maps at the level of C5 and L4, respectively (Figure 1). As a standard, 10 axial slices were segmented for each muscle group.

Reproducibility of the segmentation method was described in previous publications. The intraclass correlation coefficient (ICC) for intra-reader and inter-reader reliability was reported to be 0.966 and 0.942 at C5 level, respectively [2]. Further, an excellent reproducibility was reported for PDFF measurements at L5 level, with a root mean square absolute precision error of 0.48% [21].

### 2.4. Texture Analysis

Based on the gray-level distribution in an image, TA can be utilized to characterize structural properties of segmentations via the quantification of different texture features (TFs) [25,26,27]. TFs were calculated for each of the segmented muscle groups, using the PDFF maps. Extracted TFs included 3 global features (Variance(global), Skewness(global), and Kurtosis(global)) and 8 second-order features (Energy, Contrast, Entropy, Homogeneity and Correlation, calculated as described in [28]; Variance and Sum-average [29]; Dissimilarity [30]). Equations for the computed TFs can be found in the Appendix A. TFs were averaged over both sides, weighted by muscle volume, to extract bilateral TF values (e.g., Variance (global)_CE_, Variance (global)_ES_, and Variance (global)_PS_).

Intensity histogram analysis was applied for the calculation of global features. The optimal histogram bin size and number is a point of discussion, and strongly depending on data characteristics and purpose of the histogram analysis. In our study, we chose to calculate the number of bins by taking the median of the three following methods: Sturges’ method, Scott’s method, and the Freedman–Diaconis method. Using this approach, we obtained the most reasonable results compared to visual inspection of the histograms and best representation of the relevant data characteristics [31,32,33].

Gray-level co-occurrence matrix (GLCM) analysis was applied for the calculation of second-order TFs [28]. The preprocessing included gray-level quantization of the PDFF maps to prevent sparseness by normalizing the image intensities. For this purpose, we used 200 equally sized bins and the minimum and maximum gray levels, which correspond to values of 0% and 100%, respectively.

The entries of the GLCMs at different angular directions *θ* = (0°, 45°, 90°, and 135°) were generated by computing the joint probability of two adjacent voxel intensities at a given offset *d* = (*dx*, *dy*, *dz*) and given *θ*, with *dx, dy* and *dz* denoting the displacement along the *x*-, *y*- and *z*-axis, respectively.

Three-dimensional GLCM analysis was conducted by computation of the co-occurrence probabilities of voxel intensities from the 26 neighbors, which are aligned in 13 directions. In this process, discretization length differences were taken into account and adjusted for. Averaging the 13 directions ensures rotation invariance. All described preprocessing steps (isotropic resampling and gray-level uniform quantization) as well as the actual TA were performed with MATLAB 2021a (MathWorks Inc., Natick, MA, USA) using a radiomics toolbox (https://github.com/mvallieres/radiomics/, accessed on 18 October 2021) [34,35,36].

### 2.5. Statistical Analysis

For the statistical analyses, SPSS (version 20.0; IBM SPSS Statistics for Windows, Armonk, NY, USA) was used. Statistical significance was considered at *p* < 0.05 (two-sided) in all conducted tests.

The Kolmogorov–Smirnov test indicated non-Gaussian data distribution. Differences in age, BMI, mean PDFF, and TFs of all muscle compartments between males and females were assessed with Wilcoxon–Mann–Whitney tests. Differences in mean PDFF and TFs between the three muscle compartments were analyzed with Wilcoxon signed-rank tests. Furthermore, partial correlations (r), adjusting for age and BMI, were determined in a pairwise manner for mean PDFF and TFs between different muscle groups. For correlation testing, the whole included cohort as well as males and females were calculated separately.

## 3. Results

### 3.1. Study Population

Seventy-nine volunteers (25 men and 54 women) participated in the present study (mean age ± standard deviation: men: 43.7 ± 24.6 years; women: 37.1 ± 14.0 years). Neither age (*p* = 0.280) nor BMI (mean BMI ± standard deviation: men: 24.1 ± 6.5 kg/m^2^; women: 24.2 ± 4.8 kg/m^2^; *p* = 0.411) differed significantly between men and women (Table 1).

### 3.2. Gender-Specific Results

Mean PDFF showed significant differences between men and women in ES (*p* < 0.0001), but not in CE (*p* = 0.149) and PS (*p* = 0.611).

**Figure 2:**
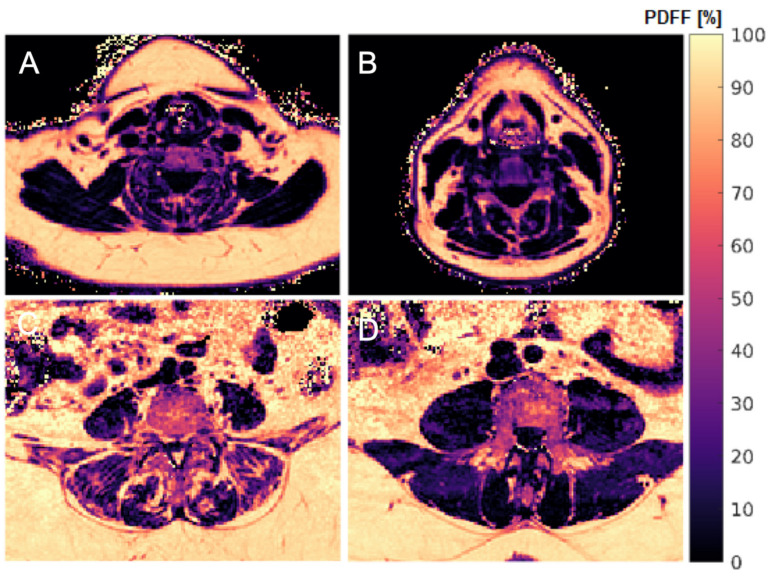
Representative color-coded PDFF maps. (**A**,**C**): 68-year-old female (PDFF_CE_ = 22.9%, PDFF_ES_ = 40.0%, PDFF_PS_ = 12.7%, BMI = 39.1 kg/m^2^). (**B**,**D**): 47-year-old male (PDFF_CE_ = 23.4%, PDFF_ES_ = 14.8%, PDFF_PS_ = 8.2%, BMI = 30.4 kg/m^2^). In the female subject, structural heterogeneity of the ES (**C**) is depicted exemplarily. PDFF, proton density fat fraction; CE, cervical muscles; ES, erector spinae muscles; PS, psoas muscles; BMI, body mass index.

There were multiple TFs with significant differences between men and women in all muscle groups. Amongst others, Variance (global), Kurtosis (global), and Dissimilarity showed significant differences between men and women in all included muscle groups (Table 1). For instance, Variance (global) demonstrated significantly higher values in men than in women in all included muscle groups (*p* < 0.001). In contrast to mean PDFF, gender-specific differences in CE and PS could be detected for Kurtosis (global) and Dissimilarity (Figure 2).

### 3.3. Muscle-Specific Results

The mean PDFF values (*p* < 0.001), Energy (*p* < 0.001), Entropy (*p* = 0.01), Homogeneity (*p* < 0.001), Sum-average (*p* = 0.012) and Correlation (*p* = 0.037) differed significantly for all three muscle groups. There were also other TA, such as Kurtosis (global), Skewness (global) and Dissimilarity, which only differed in two muscle departments. In general, a high muscle specificity for the included TA with regard to all included muscle groups could be detected.

### 3.4. Correlations of PDFF Measurements and Texture Features between Muscle Groups

**Figure 3:**
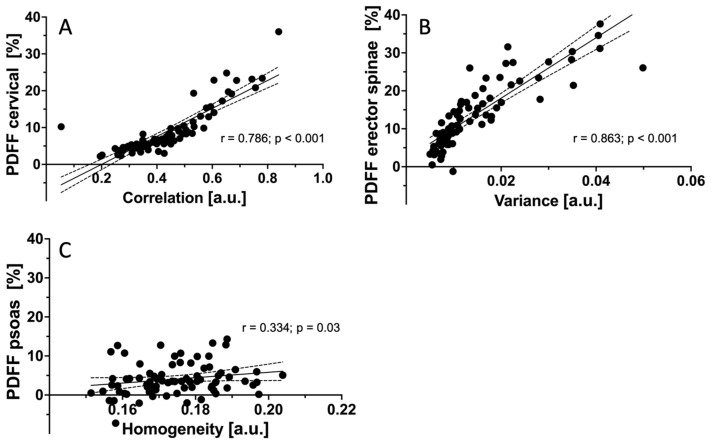
Correlations r of mean PDFF measurements and TFs after adjustment for age and BMI. Shown are the TFs with the highest r for each muscle group. (**A**): Correlation between PDFF_CE_ and the TF Correlation (r = 0.786; *p* < 0.001). (**B**): Correlation between PDFF_ES_ and the TF Variance (r = 0.863; *p* < 0.001). (**C**): Correlation between PDFF_PS_ and the TF Homogeneity (r = 0.334; *p* = 0.03). r, Pearson correlation coefficient; PDFF, proton density fat fraction; TF, texture feature; BMI, body mass index; CE, cervical muscles; ES, erector spinae muscles; PS, psoas muscles.

A partial correlation analysis, adjusted for age and BMI as potential confounders, was performed. No significant correlations could be detected regarding mean PDFF or TFs between the muscle groups in the whole cohort as well as for males and females separately. The TFs of each muscle group correlated with the extracted mean PDFF of the same muscle group, but not with the TFs of other muscle compartments. For CE, the TF correlation showed the highest correlation with mean PDFF (r = 0.786; *p* < 0.001). For ES, higher values in Variance (global) were associated with increased mean PDFF values (r = 0.863; *p* < 0.001). For PS, mean PDFF correlated significantly with Homogeneity (r = 0.334; *p* = 0.03; Figure 3).

## 4. Discussion

In the present study, the structural composition of the cervical and lumbar paravertebral musculature was assessed using the TA of PDFF maps generated with CSE-MRI. TA enabled the detection of gender-specific differences beyond the mean PDFF. In all muscle groups, PDFF heterogeneity as assessed by TA was greater in males than females. Within each muscle group, there were several TFs that correlated with the mean PDFF, but no significant inter-muscular correlations could be revealed. This finding suggests quite distinct anatomical location-specific structural patterns of the paraspinal muscles studied. Further, Variance (global) and Kurtosis (global) showed the potential for differentiating male and female structural patterns for the CE and PS, and thereby offered morphological information which could not be detected using PDFF values alone.

For a non-invasive quantitative multi-parametric muscle status assessment, CSE-MRI, MRS, and CT can be used in a preclinical but also in a clinical setting, providing valid biomarkers such as the mean PDFF and fatty acid characterization [9,11,37]. Recently, the literature on structural characterization of musculature using TA of PDFF maps has become available [19,38]. In these studies, an association of quantitative multi-parametric muscle imaging and isometric strength was reported [28,38]. Due to an aging population and a predominantly sedentary lifestyle, alterations in muscle composition, such as increasing fatty infiltration, with associated frailty and impaired functional integrity, are valuable markers of functional muscle integrity and may allow an early diagnosis of impaired function and prediction of adverse health consequences such as frailty [39]. Detecting and quantifying structural changes in the initial stages but also differentiating pathologic alterations from age-related degeneration will become a challenge in the near future.

In all three muscle compartments, the TFs Variance (global), Kurtosis (global), and Dissimilarity differed significantly in all three muscle compartments between males and females. These results are in line with prior studies showing the gender-associated structural variations in paravertebral musculature [14,15]. Our findings point to distinct intra- and inter-individual TF distribution patterns and suggest different underlying pathophysiological mechanisms for different muscle groups. By circumscribing the physiological spectrum of TF distribution in healthy subjects, our study is a first step towards the quantitative visualization of the pathophysiologic processes occurring in muscle tissue due to aging and related to gender. As our findings demonstrate, TA offers in-depth insights into paravertebral muscle composition beyond simply quantifying the extent of fatty infiltration. Thus, the present results bring us closer to understanding the intra- and inter-individual variations with regard to differentiating the start of disease manifestations from normal physiological conditions.

To the best of our knowledge, this is the first study to show gender-specific differences in paravertebral muscle composition using TFs in a considerably large cohort of healthy subjects. The results extracted from our analysis could be used in oncologic patients at risk of sarcopenia or cachexia to improve the early detection and monitoring of these severe complications and to facilitate adequate intervention [4,8,40,41]. The comparably short scan time of about two minutes per acquisition for the lumbar spine could be added to the scanning protocol in the course of oncologic disease monitoring without significant additional time expense. Altered TFs could be indicative of the start of structural changes before volumetric changes or functional impairments are evident. In the light of the potential clinical implementation in primary and secondary preventions, the reported data might serve as a reference standard for early pathology detection at the muscle level.

There are some methodological and conceptional limitations to our study. First, the retrospective nature of the study design makes it prone to selection bias. Second, manual segmentation always holds the possibility for inaccuracies. However, freely available and reliable automated segmentation algorithms are just about to be implemented for analyses of paraspinal muscles. Third, although a wide range of different age groups was recruited, we had a 2:1 female-to-male ratio, which has to be considered when interpreting our results. Fourth, there were no follow-up scans, which would have allowed a longitudinal analysis. Further longitudinal and interventional studies could also reveal changing muscle composition in association with exercise or caloric restriction. Lastly, the analysis lacks information about potential confounding factors such as physical activity. Future analyses should include information on such confounders.

## 5. Conclusions

The results of the present study demonstrate gender-specific and anatomical location-specific, distinct differences of fatty paraspinal muscle infiltration in a cohort of healthy subjects. The missing inter-muscular correlations after partial correlation testing suggest increasing BMI and age to be driving forces for muscle tissue changes. Thus, the presented TFs provide additional structural information of different muscle groups with potential for clinical implementation in differentiating physiologic from pathologic alterations.

## Figures and Tables

**Table 1:** Subject characteristics (age and BMI), PDFF values, and TFs in men (*n* = 25) and women (*n* = 54).

	Sex	Mean	SD	*p*
age	men	43.7	24.6	n.s.
	women	37.1	15.0	
BMI	men	24.1	6.5	n.s.
	women	24.2	4.8	
PDFF_cervical_	men	7.9	7.2	n.s.
	women	9.5	6.1	
PDFF_erector spinae_	men	7.4	5.6	<0.001
	women	16.9	9.2	
PDFF_psoas_	men	3.3	4.4	n.s.
	women	4.4	3.8	
Variance (global)_cervical_	men	69.2	8.7	<0.001
	women	55.0	8.4	
Variance (global)_erector spinae_	men	135.2	13.9	<0.001
	women	117.0	13.3	
Variance (global)_psoas_	men	98.6	13.6	<0.001
	women	67.1	9.8	
Skewness (global)_cervical_	men	−0.65	0.9	n.s.
	women	−0.29	0.8	
Skewness (global)_erector spinae_	men	0.13	0.9	0.005
	women	0.68	0.5	
Skewness (global)_psoas_	men	−0.68	0.3	n.s.
	women	−0.50	0.5	
Kurtosis (global)_cervical_	men	3.3	1.7	0.008
	women	2.3	1.4	
Kurtosis (global)_erector spinae_	men	3.2	0.8	<0.001
	women	1.9	1.6	
Kurtosis (global)_psoas_	men	1.1	0.5	0.006
	women	1.6	0.7	
Energy_cervical_	men	0.018	0.001	0.011
	women	0.011	0.001	
Energy_erector spinae_	men	0.001	0.0002	0.012
	women	0.0008	0.0004	
Energy_psoas_	men	0.0004	0.0001	0.039
	women	0.0005	0.0001	
Contrast_cervical_	men	468.3	89.4	n.s.
	women	528.8	248.8	
Contrast_erector spinae_	men	336.7	50.0	<0.001
	women	410.0	90.4	
Contrast_psoas_	men	391.9	51.8	n.s.
	women	391.2	78.2	
Entropy_cervical_	men	10.8	0.9	0.010
	women	11.4	0.9	
Entropy_erector spinae_	men	11.3	0.5	<0.001
	women	11.9	0.8	
Entropy_psoas_	men	12.1	0.2	n.s.
	women	11.9	0.3	
Homogeneity_cervical_	men	0.25	0.04	0.008
	women	0.22	0.04	
Homogeneity_erector spinae_	men	0.22	0.02	0.006
	women	0.20	0.02	
Homogeneity_psoas_	men	0.16	0.1	0.003
	women	0.17	0.1	
Correlation_cervical_	men	0.4	0.2	n.s.
	women	0.5	0.1	
Correlation_erector spinae_	men	0.5	0.1	<0.001
	women	0.6	0.1	
Correlation_psoas_	men	0.5	0.1	n.s.
	women	0.5	0.1	
Variance_cervical_	men	0.11	0.01	n.s.
	women	0.13	0.01	
Variance_erector spinae_	men	0.01	0.001	<0.001
	women	0.02	0.001	
Variance_psoas_	men	0.01	0.001	n.s.
	women	0.01	0.001	
Sum-average_cervical_	men	0.00241	0.0003	n.s.
	women	0.00231	0.0003	
Sum-average_erector spinae_	men	0.00208	0.0002	n.s.
	women	0.00212	0.0002	
Sum-average_psoas_	men	0.00256	0.0002	n.s.
	women	0.00240	0.0002	
Dissimilarity_cervical_	men	11.6	1.8	0.009
	women	13.4	3.7	
Dissimilarity_erector spinae_	men	10.9	3.2	<0.001
	women	12.6	2.1	
Dissimilarity_psoas_	men	13.4	0.9	0.043
	women	12.9	1.1	

## Data Availability

The datasets generated during and/or analyzed during the current study are available from the corresponding author on reasonable request.

## Appendix A

### Appendix A.1. Global Texture Features

Let *P* define the first-order histogram of a volume *V(x, y, z)* with isotropic voxel size. *P(i)* represents the number of voxels with gray-level *i*, and *N_g_* represents the number of gray-level bins set for *P*. The *i^th^* entry of the normalized histogram is then defined as:

(A1) $$p\left(i\right)=\frac{P\left(i\right)}{\sum_{i=1}^{N_{g}}P\left(i\right)}$$

The Global texture features are then defined as:

(A2) $$Variance_{global}=\sigma^{2}=\overset{N_{g}}{\underset{i=1}{\sum }}{\left(i-\mu \right)}^{2}p\left(i\right)$$

(A3) $$Skewness_{global}=\sigma^{-3}=\overset{N_{g}}{\underset{i=1}{\sum }}{\left(i-\mu \right)}^{3}p\left(i\right)$$

(A4) $$Kurtosis_{global}=\sigma^{-4}=\overset{N_{g}}{\underset{i=1}{\sum }}[{\left(i-\mu \right)}^{4}p\left(i\right)]-3$$

### Appendix A.2. Second-Order Texture Features

Let *P* define the gray-level co-occurrence Matrix (GLCM) of a quantized volume *V(x, y, z)* with isotropic voxel size. *P(i, j)* represents the number of times the voxels of gray-level *i* were neighbors with voxels of gray-level *j* in *V*, and *N_g_* represents the pre-defined number of quantized gray-levels set in *V*. The entry *(i, j)* of the normalized GLCM was defined as:

(A5) $$p\left(i,j\right)=\frac{P\left(i,j\right)}{\sum_{i=1}^{N_{g}}\sum_{j=1}^{N_{g}}P\left(i,j\right)}$$

The following quantities were also defined:

(A6) $$\mu_{i}=\overset{N_{g}}{\underset{i=1}{\sum }}i\overset{N_{g}}{\underset{j=1}{\sum }}p\left(i,j\right)$$

(A7) $$\mu_{j}=\overset{N_{g}}{\underset{j=1}{\sum }}j\overset{N_{g}}{\underset{i=1}{\sum }}p\left(i,j\right)$$

(A8) $$\sigma_{i}=\overset{N_{g}}{\underset{i=1}{\sum }}\left(i-\mu_{i}\right)2\overset{N_{g}}{\underset{j=1}{\sum }}p\left(i,j\right)$$

(A9) $$\sigma_{j}=\overset{N_{g}}{\underset{j=1}{\sum }}\left(j-\mu_{j}\right)2\overset{N_{g}}{\underset{i=1}{\sum }}p\left(i,j\right)$$

The second-order texture features are then defined as:

(A10) $$Energy=\overset{N_{g}}{\underset{i=1}{\sum }}\overset{N_{g}}{\underset{j=1}{\sum }}\left[p\left(i,j\right)\right]2$$

(A11) $$Contrast=\overset{N_{g}}{\underset{i=1}{\sum }}\overset{N_{g}}{\underset{j=1}{\sum }}{\left(i-j\right)}^{2}\;p\left(i,j\right)$$

(A12) $$Entropy=-\overset{N_{g}}{\underset{i=1}{\sum }}\overset{N_{g}}{\underset{j=1}{\sum }}p\left(i,j\right)\log_{2}\left[p\left(i,j\right)\right]$$

(A13) $$Homogeneity=\overset{N_{g}}{\underset{i=1}{\sum }}\overset{N_{g}}{\underset{j=1}{\sum }}\frac{p\left(i,j\right)}{1+\left|i-j\right|}$$

(A14) $$Correlation=\overset{N_{g}}{\underset{i=1}{\sum }}\overset{N_{g}}{\underset{j=1}{\sum }}\frac{\left(i-\mu_{i}\right)\left(j-\mu_{j}\right)p\left(i,j\right)}{\sigma_{i}\sigma_{j}}$$

(A15) $$Variance=\frac{1}{N_{g}2}\overset{N_{g}}{\underset{i=1}{\sum }}\overset{N_{g}}{\underset{j=1}{\sum }}[{\left(i-\mu_{i}\right)}^{2}p\left(i,j\right)+\left(j-\mu_{j}\right)2p\left(i,j\right)]$$

(A16) $$Sum\;average=\frac{1}{N_{g}2}\overset{N_{g}}{\underset{i=1}{\sum }}\overset{N_{g}}{\underset{j=1}{\sum }}\left[i\;p\left(i,j\right)+j\;p\left(i,j\right)\right]$$

(A17) $$Dissimilarity=\overset{N_{g}}{\underset{i=1}{\sum }}\overset{N_{g}}{\underset{j=1}{\sum }}\left|i-j\right|\;p\left(i,j\right)$$
